# Tweets, Apps, and Pods: Results of the 6-Month Mobile Pounds Off Digitally (Mobile POD) Randomized Weight-Loss Intervention Among Adults

**DOI:** 10.2196/jmir.1841

**Published:** 2011-12-20

**Authors:** Gabrielle Turner-McGrievy, Deborah Tate

**Affiliations:** ^1^Department of Health Promotion, Education, and BehaviorUniversity of South CarolinaColumbia, SCUnited States; ^2^Department of NutritionDepartment of Health Behavior and Health EducationUniversity of North Carolina at Chapel HillChapel Hill, NCUnited States

**Keywords:** Weight loss, obesity, podcasts as topic, social support

## Abstract

**Background:**

Previous interventions have shown promising results using theory-based podcasts to deliver a behavioral weight-loss intervention.

**Objective:**

The objective of our study was to examine whether a combination of podcasting, mobile support communication, and mobile diet monitoring can assist people in weight loss.

**Methods:**

In this 6-month, minimal contact intervention, overweight (n = 96, body mass index 32.6 kg/m^2^) adults were recruited through television advertisements and email listservs and randomly assigned to Podcast-only or Podcast+Mobile groups. Both groups received 2 podcasts per week for 3 months and 2 minipodcasts per week for months 3–6. In addition to the podcasts, the Podcast+Mobile group was also instructed to use a diet and physical activity monitoring application (app) on their mobile device and to interact with study counselors and other participants on Twitter.

**Results:**

Weight loss did not differ by group at 6 months: mean –2.7% (SD 5.6%) Podcast+Mobile, n = 47; mean –2.7% (SD 5.1%) Podcast, n = 49; *P* = .98. Days/week of reported diet monitoring did not differ between Podcast+Mobile (mean 2.3, SD 1.9 days/week) and Podcast groups (mean 1.9, SD 1.7 days/week; *P* = .28) but method of monitoring did differ. Podcast+Mobile participants were 3.5 times more likely than the Podcast group to use an app to monitor diet (*P* = .01), whereas the majority of Podcast participants reported using the Web (14/41, 34%) or paper (12/41, 29%). There were more downloads per episode in the Podcast+Mobile group (1.4/person) than in the Podcast group (1.1/person; *P* < .001). The number of podcasts participants reported downloading over the 6-month period was significantly moderately correlated with weight loss in both the Podcast+Mobile (*r* = –.46, *P* = .001) and the Podcast (*r* = –.53, *P* < .001) groups. Podcast+Mobile participants felt more user control at 3 months (*P* = .02), but not at 6 months, and there was a trend (*P* = .06) toward greater elaboration among Podcast+Mobile participants. There were significant differences in reported source of social support between groups. More Podcast participants relied on friends (11/40, 28% vs 4/40, 10%; *P* = .045) whereas Podcast+Mobile participants relied on online sources (10/40, 25% vs 0/40; *P* = .001).

**Conclusions:**

Results confirm and extend previous findings showing a minimally intensive weight-loss intervention can be delivered via podcast, but prompting and mobile communication via Twitter and monitoring app without feedback did not enhance weight loss.

**Trial Registration:**

Clinicaltrials.gov NCT01139255; http://clinicaltrials.gov/ct2/show/NCT01139255 (Archived by WebCite at http://www.webcitation.org/625OjhiDy)

## Introduction

The latest figures reveal that 68% of US adults are overweight or obese (body mass index [BMI] >25 kg/m^2^), with the prevalence of obesity among adult women at 35.5% and among adult men at 32.2% [1]. Overweight and obesity is associated with several chronic diseases, including type 2 diabetes, hypertension, cardiovascular disease, arthritis, hyperlipidemia, and asthma [2,3]. Even modest weight loss—around a 5% decrease in body weight—has been shown to have significant impacts in the decrease of chronic disease risk [4,5].

Behavioral interventions that target improvements in diet and physical activity are an effective way to help people lose weight and decrease chronic disease risk factors [6]. Behavioral weight-loss research programs typically involve weekly or twice-monthly, face-to-face behavioral sessions involving a group of 10–20 members and a team of weight-loss research staff [7]. Although this can be an effective way to help people lose weight, it is time and resource intensive and not easy to disseminate. Additionally, many people feel that participation in face-to-face weight-loss interventions is time consuming and often inconvenient [8]. Moving away from a face-to-face setting to a mobile delivery method is a promising strategy in the delivery of a behavioral weight-loss intervention.

Mobile technologies, such as Internet-capable mobile devices (eg, iPhone and BlackBerry), could prove to be a useful conduit for delivery of a weight-loss program. The use of mobile devices has been on the rise. In 2010, 40% of adults in the United States used a mobile phone to access the Internet or send an email or instant message, and rates of these activities increased over the previous year [9]. US adults are also accessing audio using portable devices (MP3 players), with 46% of US adults reporting they own an MP3 player [9] and 19% of Internet users reporting that they have downloaded a podcast (an audio file that can be listened to on a computer or mobile media player) [10]. Podcasts may also see a growth in use due to the popularity of cloud computing (publishing, hosting, and accessing data all online), which has made the ability to listen to and create podcasts easier [11].

Research has been emerging on the use of mobile technologies to help people achieve a healthy weight. Mobile devices have been used successfully to provide dietary guidance [12] and self-monitor weight and other health-related variables [13,14] and dietary intake [15]. Few studies, however, have used an entirely mobile device-based approach to deliver a behavioral weight-loss intervention. For example, studies may use text messaging sent to mobile devices (short message service [SMS]) for support in addition to face-to-face group sessions [16]. One study that used an entirely mobile-based approach was conducted by Patrick and colleagues [17] among overweight men and women. This behavioral weight-loss intervention was delivered entirely through SMS with a mixture of standard and targeted messages to participants, who on average had lost 3.16% of their body weight at 4 months [17]. Another technology-delivered intervention that contained a sizable mobile component targeted increasing physical activity among healthy, slightly overweight (BMI 26.3 kg/m^2^) men and women. Participants in the intervention group received targeted messages on overcoming barriers via the Internet and reminders to be physically active delivered via SMS and email, and had access to a message board to discuss experiences with other study participants [18].

Our previous work demonstrated that a short-term behavioral weight-loss intervention could be successfully delivered via podcast [19]. In that 3-month trial, 78 participants were randomly assigned to receive a podcast designed based on social cognitive theory [20] (enhanced) or a popular weight-loss podcast (control) available online. Weight losses were significantly greater in the enhanced podcast group (mean –2.9, SD 3.5 kg) than in the controls (mean –0.3, SD 2.1 kg; *P* < .001 between groups). This study, however, was short-term and weight losses were modest. A podcast-only format also limited the ability to provide participants with easy ways to self-monitor diet and physical activity and to receive social support (that would normally be delivered in a face-to-face group setting). To our knowledge, no previous studies have employed a combination of podcasting, mobile support communication, and mobile diet monitoring to assist people in weight loss. Therefore, we explored the use of this enhanced mobile approach as a way to help people lose weight in the Mobile Pounds Off Digitally (Mobile POD) intervention.

## Methods

### Study Population and Measures

Overweight and obese men and women (BMI 25–45 kg/m^2^, 18–60 years old) were recruited through television advertisements and email listservs in the Raleigh-Durham, North Carolina, USA metropolitan area for this 6-month randomized trial. Participants were excluded if they smoked, had an unstable medical status or uncontrolled thyroid condition, were unable to attend the 3 monitoring visits or increase walking as a form of exercise, had a psychiatric illness, were in treatment for alcohol or drug dependency, had an eating disorder, were currently participating in a weight-loss program, or were pregnant, breastfeeding, or planning on becoming pregnant within the next 6 months. Participants were also required to be able to complete the Physical Activity Readiness Questionnaire [21] and were excluded for a history of myocardial infarction or stroke, and had to obtain physician consent for participation if endorsing yes on other items (such as use of hypertensive medications or bone and joint issues). Participants were required to have access to a body weight scale for self-monitoring weight and had to own one of four types of Internet-capable mobile devices: iPhone, iPod Touch, BlackBerry, or an Android-based phone. Participants were required to have access to the Internet and be comfortable using a computer. The University of North Carolina at Chapel Hill Institutional Review Board approved the study, and all the participants gave written informed consent. Participants received a US $20 incentive payment for completing all 3- and 6-month assessment activities.

Study research assistants screened participants. On meeting screening criteria, participants were invited to an orientation session where they learned more about the study, were shown how to complete online baseline questionnaires, and filled out the consent form. Participants were then given 2 weeks to complete questionnaire items (all completed online) assessing the following: demographics; dietary intake from 2 days of unannounced 24-hour dietary recalls (1 weekday and 1 weekend day) collected using the Automated Self-administered 24-hour Dietary Recall [22]; physical activity (Paffenbarger Physical Activity Questionnaire) [23]; self-efficacy (Weight Efficacy Life-Style Questionnaire [WEL]) [24]; and eating behaviors (Eating Behavior Inventory [EBI]) [25]. Participants were also asked (on a 1- to 7-point Likert scale) to rate how supported they felt in their weight-loss efforts at 6 months. These questionnaire items were transformed from paper instruments to online methods (but had not been previously validated for online use).

Participants were randomly assigned using a computerized random numbers generator (as conducted by study investigators) once they completed of all their baseline questionnaires. A face-to-face group visit was scheduled to obtain objective height and weight. Before participants were given their randomization assignment, their weight was measured in light street clothes using a calibrated Tanita BWB-800 digital scale (Tanita, Arlington Heights, IL, USA) accurate to 0.1 kg. Height was measured using a wall-mounted stadiometer measured (Perspective Enterprises, Portage, MI, USA) after participants had removed hats and shoes. Once all baseline measures were collected, participants were given an overview of which group they were randomly assigned to and were provided with more details about group assignment. Both conditions were active treatments and participants were not told which group was the intervention of interest or enhanced group. Neither study participants nor investigators were blind to treatment assignment.

### Intervention and Control Conditions

Participants could be randomly assigned to one of two conditions: podcast-only (Podcast) or podcast plus enhanced mobile media intervention (Podcast+Mobile). Both groups received 2 podcasts per week for 3 months (approximately 15 minutes each) and 2 minipodcasts per week for months 3–6 (approximately 5 minutes each). Participants had access to a group-specific podcast site, where they could subscribe to the podcast using their mobile device or listen directly to the podcast on a computer. The content and design of the podcasts have been described elsewhere [19]. Briefly, podcasts were designed using constructs from social cognitive theory [20]. Podcasts were written and recorded prior to the start of the study. Podcasts delivered in the first 3 months contained a section on nutrition and physical activity information, an audio blog of a man or a woman trying to lose weight, a soap opera, and a goal-setting activity. Podcasts delivered in months 3–6 contained only the nutrition and exercise portion of the podcast and focused on overcoming barriers and problem-solving issues. The Podcast group—but not the Podcast+Mobile group—received a book with calorie and fat gram amounts of food to assist them in monitoring their dietary intake. In addition to the podcasts, the Podcast+Mobile group was also instructed to download a diet and physical activity monitoring application (app) (2010 version of FatSecret’s Calorie Counter app, FatSecret.com, which released additional updates in January 2011) and a social networking site’s (Twitter) app to their mobile device (both free for download). Participants created a user account on Twitter, were told to log on (through either their mobile device or their computer) to Twitter at least once daily to read messages posted from the study coordinator, and were encouraged themselves to post at least daily to Twitter. Participants could choose any user name they wanted (to protect their identity) and were instructed on how to make their Twitter account private (if they chose to do so). During months 0–3, Podcast+Mobile participants were divided into 4 groups to create Twitter cohorts of 11–12 people. They were sent a list of everyone’s Twitter user names within their cohort, were instructed to follow everyone in their cohort, and were reminded to send follow requests to participants and to accept requests until everyone in each cohort was following one another. During months 3–6, Podcast+Mobile participants were asked to follow everyone in the study, and similar procedures were used to allow everyone within the Podcast+Mobile group to follow one another. The study coordinator sent out 2 messages per day to the group, which reinforced messages from the podcasts, posed questions to the group to facilitate discussion, and encouraged participants to share tips and recipes with one another that would assist in weight loss. Such messages were prompts to attend to weight-loss behavior, and encouraged communication but were not individualized. The study coordinator did not participate in discussions initiated by participants. All participants received information on safe exercise practices.

### Assessment Periods

Change in body weight was the main outcome of the study, and body weight was collected at baseline, 3 months, and 6 months at the study site. In addition to the diet, physical activity, and psychosocial measures discussed above, other measures were collected at both 3 and 6 months including novelty, cognitive load [26], user control [27], elaboration (Elaboration Likelihood Model Questionnaire) [28], and process evaluation questions, all via online questionnaire. Participants were also sent a weekly online questionnaire link so they could report the number of podcasts they had listened to that week, their weight, number of days they monitored their diet and physical activity, and, for the Podcast+Mobile group, questionnaire items assessing use of Twitter. Participants who did not complete their weekly questionnaire were sent an email reminder and received a phone reminder after 2 weeks in a row of missed weekly questionnaire submissions. The number of Twitter messages per participant was also recorded over the course of the study, and an objective measure of number of downloads per podcast by treatment group was obtained from the podcast hosting site.

### Statistical Analyses

Power calculations for the study used the 3-month weight loss from the previous podcasting study [19] as compared with the 3-month weight loss seen in a Web-based study that used an automated email feedback group (similar to our Podcast+Mobile group) [29]. This resulted in an effect size of *r* = .2934 and a Cohen *d* of 0.6138. Sample size per intervention arm for 2-sided tests of significance at alpha = .05 and power 1 – beta = 80% would be 43 per group (86 total required N). To account for attrition, we attempted to recruit 95–105 total participants.

We conducted all data collection and analyses using intention-to-treat by using imputation (baseline observation carried forward), with the exception of some variables that we collected only at 6 months (such as information processing variables), which we assessed using completers only. Between-subjects *t* tests were calculated for differences between continuous variables, and paired-samples *t* tests were used to examine differences within groups. Logistic regression models were used to assess demographic predictors of study discontinuation at 6 months. Demographic information that contained multiple categories (such as education, ethnicity, and marital status) was dichotomized and the chi-square test of independence was used to assess differences between groups at baseline. Analysis of variance was used to examine mean differences within 3 or more groupings, and repeated-measures analysis of variance was used to assess changes over time among the continuous variables. Pearson correlation was used to examine the relationship between number of podcasts downloaded and the number of Twitter posts with weight loss. All analyses were conducted using SPSS 16.0 for Windows software (IBM Corporation, Somers, NY, USA) with a *P* value of .05 used to indicate statistically significant differences.

## Results

**Figure 1:**
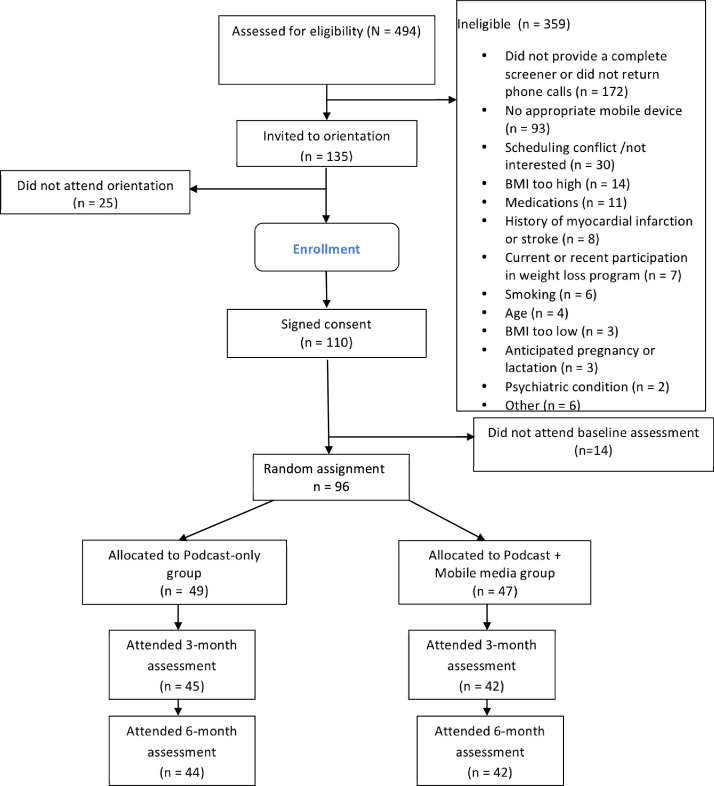
Eligibility, enrollment, random assignment, and assessment of study participants. BMI = body mass index.

Participants were recruited from July 2010 to August 2010, and the study ran until February 2011. Figure 1 shows the flow of participants through recruitment, intervention, and follow-up. Of the 494 volunteers who inquired about the study, 359 (72.7%) were ineligible and 135 were invited to an orientation, of whom 96 enrolled in the study. Table 1 outlines baseline demographics. There were no significant differences in baseline demographics between the two groups. More people in the Podcast+Mobile group than in the Podcast group reported previously downloading a health-related podcast (*P* = .04) or installing a healthy diet-related app to their mobile device (*P* = .04). In a model examining demographic factors as predictors of noncompletion of the study at 6 months, there was a significant effect of age (*P* = .005) and a trend with ethnicity (*P* = .06), but not of gender, group assignment, or baseline BMI. Participants who did not complete the study at 6 months (n = 10) were younger (mean 31.0, SD 11.2 vs 44.3, SD 10.3 years) and were 4.7 times (95% confidence interval, 0.96–23.24) less likely to be white (4/10, 40% black, 2/10, 20% Asian, and 4/10, 40% white).

**Table 1:** Baseline demographic data for Podcast-only and enhanced Podcast+Mobile group participants

		Podcast group (n = 49)	Podcast+Mobile group (n = 47)
Age (years), mean (SD)		43.2 (11.7)	42.6 (10.7)
**Sex, n (%)**			
	Male	13 (27)	11 (23)
	Female	36 (73)	36 (77)
**Race/ethnicity, n (%)**			
	Black	10 (20)	9 (19)
	White	38 (78)	35 (75)
	Other	1 (2)	3 (6)
**Hispanic, n (%)**			
	Yes	0	0
	No	49 (100)	47 (100)
**Marital status, n (%)**			
	Not married	23 (47)	16 (34)
	Married	26 (53)	31 (66)
**Education, n (%)**			
	College or less	19 (39)	24 (51)
	Graduate degree	30 (61)	23 (49)
**Type of Internet-capable mobile device, n (%)**			
	iPhone	14 (29)	18 (38)
	iPod Touch	13 (26)	13 (28)
	BlackBerry	18 (37)	11 (23)
	Android-based phone	4 (8)	5 (11)
Body mass index (kg/m^2^), mean (SD)		32.2 (4.5)	32.9 (4.8)
Number of years participant has owned Internet-capable mobile device, mean (SD)		1.6 (1.2)	1.3 (0.8)
Number of participants who were members of Twitter at baseline, n (%)		17 (35)	16 (34)
Number of participants who had previously downloaded a health-related podcast, n (%)		14 (29)^a^	23 (49)
Number of participants who had previously downloaded an application to their mobile device to help them eat better, n (%)		18 (37)^a^	27 (57)

^a^ χ^2^ test of independence *P* = .04.

**Figure 2:**
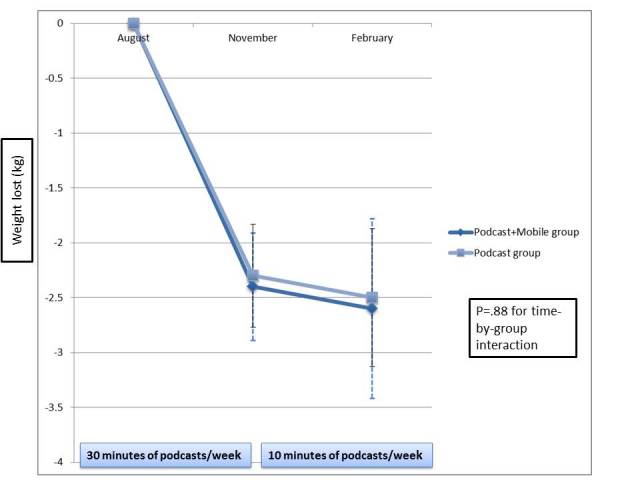
Weight (mean, SE) lost by group over 6 months.

Figure 2 details the weight loss by group over the 6-month study. The Podcast+Mobile group lost a mean of –2.4 (SD 3.4) kg at 3 months (vs –2.3, SD 3.3 kg in the Podcast group) and an additional –0.2 (SD 3.0) kg from months 3 to 6 (vs –0.3, SD 1.8 kg in the Podcast group; *P* = .88 for time-by-group interaction). Table 2 outlines group differences in percentage weight loss, diet, physical activity, self-efficacy, knowledge, and eating behaviors. The group-by-time interaction was not significant for any of the variables. The percentage weight loss at 3 or 6 months did not differ between the groups. There were no significant differences between groups in energy expenditure or intake at 3 or 6 months. Groups did not differ in changes in fat intake, self-efficacy (WEL score), or weight-related eating behaviors (EBI score) at 3 or 6 months.

**Table 2:** Changes in weight, physical activity, dietary intake, self-efficacy, knowledge, and eating behaviors at 3 and 6 months^a^

		Podcast group (n = 49)	Podcast+Mobile group (n = 47)	Significance (*P* value)
**Weight change (%)**				
	0–3 months	–2.6 (3.8)	–2.6 (3.5)	.97
	0–6 months	–2.7 (5.1)	–2.7 (5.6)	.98
**Intentional physical activity change (caloric expenditure, in kcal)**				
	0–3 months	82.7 (153.2)	94.5 (130.2)	.68
	0–6 months	96.7 (185.5)	86.8 (182.1)	.79
**Change in energy intake (kcal)**				
	0–3 months	–146.3 (506.3)	–341.1 (612.1)	.09
	0–6 months	–242.5 (558.8)	–288.8 (553.0)	.69
**Change in fat intake (g)**				
	0–3 months	–13.6 (23.8)	–15.2 (31.0)	.78
	0–6 months	–14.5 (32.0)	–15.0 (26.4)	.92
**Change in weight-loss self-efficacy (WEL^b^ score)**				
	0–3 months	12.5 (24.4)	12.5 (29.0)	.99
	0–6 months	20.1 (26.0)	17.6 (25.3)	.64
**Change in weight-loss knowledge score**				
	0–3 months	1.2 (1.8)	0.74 (1.9)	.24
	0–6 months	1.1 (1.8)	0.66 (1.7)	.17
**Change in eating behaviors (EBI^c^ score)**				
	0–3 months	8.6 (12.6)	11.7 (11.8)	.21
	0–6 months	9.8 (11.3)	12.4 (11.4)	.27

^a^ All data are mean (SD).

^b^ Weight Efficacy Life-Style Questionnaire.

^c^ Eating Behavior Inventory.

Table 3 outlines differences in information processing variables measured at 3 and 6 months, as well as the number of podcasts downloaded and days per week diet and physical activity were self-monitored. Podcast+Mobile participants reported more user control at 3 months but not at 6 months (*P* = .08). There was no significant difference in cognitive load but Podcast+Mobile participants reported that the intervention was more novel at both 3 and 6 months. There was no significant difference in elaboration at 3 months between groups but there was a trend (*P* = .06) at 6 months with Podcast+Mobile participants reporting more elaboration than Podcast participants. There was no difference between groups at 0–3 months or 3–6 months in mean number of reported podcasts downloaded. However, the objective data from the podcast hosting site showed a significant difference in the number of downloads by group with more downloads occurring in the Podcast+Mobile group than in the Podcast group during both 0–3 months (*P* < .001) and 3–6 months (*P* < .001). The number of podcasts participants reported downloading over the 6-month period was significantly moderately correlated with weight loss in both the Podcast+Mobile (*r* = –.46, *P* = .001) and the Podcast (*r* = –.53, *P* < .001) groups. There was no difference between groups in the mean days per week that dietary intake or physical activity were self-reported by participants. The method of self-monitoring, however, differed by group. Podcast+Mobile participants were 3.5 times more likely than the Podcast group to use an app to monitor diet over the course of the study (95% confidence interval, 1.29–8.84; *P* = .01), whereas the majority of Podcast participants reported using Web (14/41, 34%) or paper (12/41, 29%) methods. Collapsing the data across groups, mean days per week of dietary self-monitoring over the 6-month study differed by method used to record intake: mean 2.9 (SD 1.9) days/week mobile app, n = 37; 2.3 (SD) 1.9 days/week website, n = 24; and 1.6 (SD 1.3) days/week paper journal, n = 17; n = 3 reported using nothing for monitoring and n = 15 did not report a method; *F*_2,7_ = 3.41, *P* = .04). There was no difference in number of days of diet monitoring or weight loss by type of mobile device. Tukey honestly significant difference (HSD) post hoc analysis shows a significant difference in mean days per week of diet self-monitoring between paper journal methods and using a mobile app to record diet (*P* = .03).

**Table 3:** Information processing variables at 3 and 6 months and podcast downloads and self-monitoring outcomes by group^a^

		Podcast group (n = 49)	Podcast+Mobile group (n = 47)	Significance (*P* value)
**User control**^b^				
	0–3 months	13.2 (5.0)	15.4 (3.5)	.02
	3–6 months	13.9 (3.9)	15.4 (4.0)	.08
**Cognitive load**^b^				
	0–3 months	11.7 (2.2)	11.5 (1.8)	.72
	3–6 months	11.2 (2.6)	11.3 (2.4)	.79
**Novelty**^b^				
	0–3 months	8.9 (3.1)	10.9 (3.0)	.01
	3–6 months	9.1 (3.5)	11.1 (2.9)	.01
**Elaboration**^b^				
	0–3 months	47.9 (10.0)	51.3 (8.4)	.1
	3–6 months	45.7 (13.0)	50.3 (8.7)	.06
**Mean number of podcasts (out of 24) participants reported they downloaded**				
	0–3 months	14.5 (7.6)	16.4 (7.2)	.20
	3–6 months	8.2 (8.6)	9.0 (9.1)	.67
**Number of downloads per podcast episode per person from podcasting host site**				
	0–3 months	1.51 (0.65)	2.00 (0.52)	<.001
	3–6 months	0.66 (0.15)	0.87 (0.20)	<.001
**Number of total podcast downloads from podcasting host site**				
	0–3 months	74.2 (31.8)	94.1 (24.6)	<.001
	3–6 months	32.5 (7.2)	40.7 (9.5)	<.001
**Mean days/week reported recording dietary intake**				
	0–3 months	2.4 (2.0)	2.9 (2.1)	.26
	3–6 months	1.3 (1.7)	1.7 (2.0)	.39
**Mean days/week reported recording physical activity**				
	0–3 months	2.6 (2.0)	2.4 (1.8)	.63
	3–6 months	1.6 (2.1)	1.5 (1.9)	.81

^a^ All data are mean (SD).

^b^ At 3 months, n = 43 for Podcast and n = 41 for Podcast+Mobile, and at 6 months n = 40 for both groups for all measures except elaboration at 6 months (n = 43 for Podcast and n = 40 for Podcast+Mobile).

Although there was no difference in how supported (rated 0–7) participants reported feeling at 6 months (Podcast+Mobile mean 5.0, SD 1.5 vs 4.8, SD 1.7; *P* = .67), there was a significant difference in the main form of social support participants reported during the 6-month trial. More of the Podcast participants reported mainly relying on friends for social support (11/40, 28% Podcast vs 4/40, 10% Podcast+Mobile; *P* = .045) and more Podcast+Mobile participants reported their main form of support came from online sources, such as Twitter, Facebook, or blogs (10/40, 25% Podcast+Mobile vs 0% Podcast; *P* = .001). Within the Podcast+Mobile group, 94% (n = 44) posted at least once to Twitter, with 64% (n = 30) posting at least weekly during the first 3 months and 28% (n = 13) posting weekly or more during months 3–6. Podcast+Mobile participants made a mean of 2.1 (SD 3.1) posts to Twitter per week, with significantly more posts being made in the first 3 months (2.8, SD 3.6 posts/week) than in months 3–6 (1.3, SD 3.0 posts/week; *P* < .001). On average, Podcast+Mobile group participants did not view Twitter as being useful to their weight-loss efforts (mean score of 3.4, SD 1.8, out of 7). Two technical issues occurred over the course of the study. FatSecret released an update to their app on January 3, 2011, which led to the app crashing for a few days before it was resolved by FatSecret. Also, on September 21, 2010, Twitter experienced a virus attack that was activated when users rolled their mouse over a blacked-out block of text [30]. It was quickly resolved by Twitter and no participant reported being affected by the virus (as it only affected Web users and not mobile app users). 

## Discussion

This study explored the 6-month efficacy of a weight-loss intervention delivered by podcast and the additive benefit of mobile diet monitoring and communication delivered via a social networking site (Twitter). The results show that a very-low-intensity intervention that is delivered entirely by mobile technology can produce short-term, modest weight loss. The addition of prompts and support provided via Twitter, as well as mobile monitoring provided via a diet and physical activity app, did not enhance weight losses over what was seen by just using a podcast alone and encouraging participants to monitor with an approach of their choice.

Most participants in this study did not achieve a 5% weight loss, which is the level thought to be clinically meaningful; however, the time of year when the study was administered may have affected the outcomes. Participants completed the 3-month follow-up a week prior to the Thanksgiving holiday (United States). Months 3–6 occurred over the holiday season, including Thanksgiving, Christmas, Hanukkah, and New Year’s. Many of the podcasts during this time discussed topics regarding holiday weight gain, such as altering holiday recipes, finding time to exercise when your schedule changes, and finding strategies to eat healthy at holiday parties. Observational studies have shown that the average weight gain over the holiday period in the United States ranges from 0.4 to 0.8 kg [31,32]. Holiday weight gain may be greater among those who are overweight or obese than among normal-weight people [31,32]. In addition, those who have lost weight are more vulnerable than normal-weight individuals to holiday weight gain and weight retention after the holiday [33]. Therefore, the fact that only minimal weight loss occurred during months 3–6 in the present study (approximately –0.25 kg or –0.55 lb) demonstrates that this intervention may have worked to prevent holiday weight gain, versus promoting additional weight loss during this vulnerable period. In addition, the podcasts during months 3–6 were shorter, only 5 minutes (10 minutes total/week), as compared with 15 minutes (30 minutes total/week) during months 0–3. It is possible that moving to a shorter intervention dose was insufficient to promote greater weight loss during this time. The combination of seasonality (holidays) and the lowering of the intervention dose at 3 months may have occurred too soon within the intervention, before participants had fully learned the behaviors needed for successful weight loss.

The intervention groups did not differ in changes in EBI score, WEL score, energy expenditure, or energy intake, demonstrating that the addition of social networking support and mobile diet and physical activity monitoring did not enhance these outcomes beyond receiving the theory-based podcast alone. Since there were no differences in days of self-reported diet monitoring by group and no differences in weight loss, these findings are not surprising. There were differences, however, in some of the information processing variables. Study interventionists delivered 2 prompt-style messages to the Podcast+Mobile group via Twitter each day. We chose Twitter as a way to deliver real-time messages to participants from study interventionists (2 messages/day), which we hypothesized would be similar in effectiveness to delivering messages via SMS [17]. We also chose this social networking site to enhance elaboration, since messages posted by study staff reinforced messages delivered in the podcasts. Although elaboration did not differ between the groups at 3 months, there was a trend (*P* = .06) at 6 months, and it is possible that the addition of Twitter messages was beneficial in reinforcing weight loss-related messages and allowing for more effective information processing [34]. In addition to enhanced elaboration at 6 months, Podcast+Mobile participants reported greater user control at 3 months. Learning occurs in a different ways, so the more control learners have over their experience, the more variety of learning styles can be accommodated [35]. Therefore, providing additional learning channels (above audio alone) may have provided an additional feeling of user control and allowed participants to feel more motivated to learn and attend to messages [27]. Participants in both groups reported low levels of cognitive load (11 out of 14; with a higher score corresponding to less cognitive load) and therefore the addition of Twitter to the intervention did not increase the burden on working memory [36]. The addition of Twitter and mobile diet monitoring led to greater feelings of novelty among Podcast+Mobile participants than for the participants who received only a podcast. This enhanced novelty among Podcast+Mobile participants may have been due to the very low reported use of Twitter (34%) among participants at baseline. Despite changes in these information processing variables, weight losses did not differ between the groups. Greater elaboration and user control, however, may have led to greater podcast usage, as evidenced by more podcast downloads to the Podcast+Mobile group site. Greater usage of weight-loss study components has been shown to lead to improved weight losses [37]. Our prior podcasting study showed greater elaboration and user control among the enhanced theory-based podcast at 3 months than among the control podcast [19]. The present study also demonstrated high elaboration and user control within the Podcast+Mobile group as compared with the Podcast group, but no differences in cognitive load, demonstrating that the additional components of Twitter and mobile diet monitoring did not increase cognitive burden and allowed for continued elaboration and user control.

It is possible that the addition of Twitter and mobile monitoring was a distraction from what was already a successful weight-loss intervention delivered by podcast only. Because weight losses were modest in our previous podcasting trial [19], we sought to add components that are common to face-to-face behavioral interventions (self-monitoring, group support, and contact with study counselors) and deliver them in a mobile manner. These components appeared to enhance user control (at 3 months) without increasing cognitive load. The self-monitoring app and Twitter, however, were poorly used by participants. This demonstrates that these additional components were not well integrated by participants. In addition, the Podcast+Mobile participants reported relying more on online sources of support than on friends and family. There may have been a negative impact of displacing support from real-life friends and family with online social networks.

Self-monitoring of dietary intake is an important component of behavioral weight-loss programs [38]. We hypothesized that the use of a mobile diet monitoring app would increase dietary self-monitoring. We saw no differences in self-monitoring days per week at either 3 or 6 months between the groups, with both groups reporting monitoring an average of approximately 2.5 days/week from 0 to 3 months and 1.5 days/week from months 3 to 6. Although days per week of diet monitoring did not differ between groups, method of monitoring did. Podcast+Mobile participants were instructed to use the FatSecret Calorie Counter app. We chose this app due to its availability on all 3 major mobile phone platforms. Only 60% (24/40) of participants in the Podcast+Mobile group, however, reported using a mobile app for diet monitoring. The Podcast group was given a book to monitor their calorie intake, but at 6 months, only 29% (12/41) of participants were using a paper recording method to self-monitor dietary intake. Podcast participants may have been at an advantage with regard to diet monitoring, since they were able to choose which method they preferred to use and, if they chose to use an app (13/41, 32% of participants), they were free to choose which one would be best for their device. In a recent study examining differences in dietary intake between participants randomly assigned to monitor their diet via a handheld electronic device or paper journal, no differences were seen between the groups in weight loss, energy intake, or percentage of energy (kcal) from fat [39]. This demonstrates that adherence to monitoring is what is important for weight loss [40], regardless of method. With both groups collapsed, we did see a significant difference in number of days per week participants reported self-monitoring diet, with participants using a mobile device recording twice as many days as those using a paper method. This finding warrants further exploration in future studies and points to the possibility of recommending self-monitoring methods that are tailored to users’ needs, mobile devices, and comfort level with technology.

The study content was delivered mainly through podcast messages for both groups. The number of podcasts participants reported downloading from months 0 to 3 and months 3 to 6 did not differ between the groups, but the objective number of downloads from each groups’ podcast site did differ significantly. There were significantly more downloads per person in the Podcast+Mobile group than in the Podcast group. This may not have corresponded to more podcasts listened to, since participants may have initiated a download of an episode but later returned to finish the episode (starting another compete download). It is possible that Podcast+Mobile participants may have listened to podcast episodes multiple times as well or shared the podcast links with friends. Since Twitter messages prompted Podcast+Mobile participants to listen to the latest podcast episode, this method may have been an additional way for participants to remember to access the podcasts or be reminded to go back and listen to an episode as a refresher.

Social support has been shown to be a possible key component in behavioral weight-loss programs [41]. The present study used Twitter as a method to deliver prompts from the program and allow participants to support each other during their weight-loss efforts. The study coordinator posted 2 messages to participants per day that could be easily automated, as the messages were not in response to participants. An additional advantage of Twitter is that it allowed for real-time support, such as asking fellow participants about healthy dining options once a participant arrives in a restaurant. However, the number of Twitter posts averaged 2 per week, and declined over time, so participants were not communicating with other group members frequently. Podcast+Mobile participants were initially assigned to a small cohort of 10–11 people. This was to allow for effective communication and to prevent participants from being overwhelmed with too many posts by other members. Participation was sporadic, however, so during the 3- to 6-month time period, Podcast+Mobile participants were asked to follow everyone within the Podcast+Mobile group to help facilitate more active discussion. It is possible there was not enough interaction to provide adequate social support, or this study may be similar to other studies that found no benefit of enhancing social support among weight-loss participants [42]. The podcasts, which were the same for both groups, encouraged establishing a good support system for weight loss. Both groups felt equally supported in their weight-loss efforts, but source of support differed. Podcast participants mainly turned to real-life friends as sources of support, whereas Podcast+Mobile participants relied on Internet-based sources, namely Twitter.

Several studies have shown that targeting dietary self-monitoring, providing social support, and emphasizing both dietary changes and physical activity are key components to successful face-to-face behavioral weight-loss programs [43]. We sought to improve on our previous trial [19] by enhancing these components through advanced mobile technology means. Both groups, however, found ways to self-monitor diet and obtain social support, meaning that structural aspects of the groups differed in type but not amount, which was reflected in the weight-loss outcomes. Several of the components of Internet-based weight-loss interventions that have been shown to be effective were part of the Podcast+Mobile intervention, including self-monitoring, use of established behavioral strategies, and social support [44]. Other effective aspects were not included, such as study counselor feedback and tailoring of messages and information [44]. Future studies examining ways to enhance weight loss of participants receiving a mobile, podcast-delivered weight-loss intervention may wish to find ways to provide more individualized feedback (which could be human or automated) on dietary self-monitoring and intake, physical activity progress, and weight loss. Additionally, ways to tailor the intervention components to participant characteristics, such as body weight, dietary intake, or physical activity, may enhance weight losses over nontailored approaches [44]. Designers of future interventions may wish to find ways to offer a multimodal intervention to appeal to diverse users.

There were several strengths to this study. This intervention used a randomized design and intention-to-treat analysis. The study also had strong retention rates, used an objective measure for weight, and included 2 unannounced days of dietary intake collected using a multiple-pass method. This intervention is an approach that could be easily disseminated, as there was very minimal contact with participants, everything was delivered remotely, and it was low cost. The study also had some limitations. The study population was mostly white and female. The study was short-term (6 months) and so it was able to examine initial weight loss but not weight-loss maintenance. The groups also differed in prior experience with apps and podcasts, showing that those in the Podcast+Mobile group may have been more technically savvy or more familiar with the technology used in the study at baseline. This did not seem to affect the results, and equal numbers of participants from each group were members of Twitter at baseline. Participants who did not complete the study were significantly younger and less likely than completers to be white, and therefore some aspect of the study may not have been well suited to this demographic group.

In summary, the Mobile POD, 6-month, minimal-contact intervention was effective at helping participants achieve a mean weight loss of 2.7% of their body weight, and perhaps was useful in preventing holiday weight gain. Both groups reported similar levels of social support and days of dietary monitoring, demonstrating that providing these components was not necessary, and that the podcasts’ emphasis on directing participants to find these components was effective. Future studies may wish to find ways to combine podcasts with tailored feedback for participants to enhance compliance with dietary and physical activity recommendations and to improve weight loss.

## Abbreviations

app: application
BMI: body mass index
EBI: Eating Behavior Inventory
Mobile POD: Mobile Pounds Off Digitally
SMS: short message service
WEL: Weight Efficacy Life-Style Questionnaire
